# Suspected Microplastic-like Particles in Human Gastric Autopsy Samples: An Exploratory Histopathological and Biochemical Study

**DOI:** 10.3390/life16091412

**Published:** 2026-08-26

**Authors:** Eugen-Toma Radu, Nastaca Alina Palade, Crina Cristina Solomon, Oana Gabriela Somcutian, Manuela Pia Pumnea, Maria Totan, Claudia Maria Mihuț, Cecilia Georgescu, Diter Atasie, Anca Maria Arseniu, Felicia Gabriela Gligor

**Affiliations:** 1 County Emergency Clinical Hospital Sibiu, 550025 Sibiu, Romania; eugen.radu@ulbsibiu.ro; 2 PreclinicalImage Department, Faculty of Medicine, Lucian Blaga University of Sibiu, 550169 Sibiu, Romania; manuela-pia.pumnea@ulbsibiu.ro (M.P.P.); maria.totan@ulbsibiu.ro (M.T.); anca.arseniu@ulbsibiu.ro (A.M.A.); felicia.gligor@ulbsibiu.ro (F.G.G.); 3 Department of Pathology, “Niculae Stancioiu” Heart Institute Cluj-Napoca, 400001 Cluj-Napoca, Romania; oanatarta15@yahoo.com; 4 Biology and Ecology Research Center, Faculty of Sciences, Lucian Blaga University of Sibiu, 550012 Sibiu, Romania; claudia.mihut@ulbsibiu.ro; 5 Faculty of Agriculture Sciences, Food Industry and Environmental Protection, Lucian Blaga University of Sibiu, 550012 Sibiu, Romania; cecilia.georgescu@ulbsibiu.ro; 6 Medlife Polisano Hospital Sibiu, 550024 Sibiu, Romania; atasie.diter@ulbsibiu.ro; 7 Department of Clinical Medicine, Faculty of Medicine, Lucian Blaga University of Sibiu, 550024 Sibiu, Romania

**Keywords:** microplastics, human tissue, stomach, polarized light microscopy, post-mortem study

## Abstract

Background: Human exposure to microplastics (MPs) is an emerging public health concern, yet their presence and pathological significance in gastric tissues remain poorly understood. This study investigated suspected MP-like particles in human gastric autopsy specimens, examining their association with histopathological changes and routine biochemical parameters. Methods: A retrospective post-mortem study was conducted on gastric tissue samples from 55 adults who underwent autopsies between 2022 and 2025. Tissue samples were digested using a hydrogen peroxide method, vacuum-filtered, stained with Nile Red, and quantified via ImageJ. Histopathological evaluation using hematoxylin–eosin staining and polarized light microscopy identified tissue-embedded suspected MP-like particles and assessed inflammation, necrosis, and fibrosis. Data were analyzed based on suspected MP-like particle detection status. Results: Suspected gastric MP-like particles were detected in 15 of 55 cases (27.3%). Most positive cases exhibited a low particle burden (median 1/g). Polarized light microscopy additionally served as a technical assessment tool, revealing birefringent structures measuring 26–84 μm within the gastric mucosa or lumen. All suspected MP-like particle-positive cases showed gastric inflammation compared to 35.0% of suspected MP-like particle-negative cases. Fibrosis and necrosis occurred exclusively in the suspected MP-like particle-positive group but lacked statistical significance. No significant associations were found between suspected MP-like particle detection and demographics, comorbidities, or systemic biochemical markers. Conclusions: Suspected gastric MP-like particles were identified in over a quarter of autopsy tissues and were strongly associated with localized gastric inflammation.

## 1. Introduction

Synthetic polymers represent one of the most transformative classes of materials of the 21st century, impacting society and daily life to an extent that cannot be overestimated [1]. Modern plastics consist of complex assemblies of base polymers—such as polyethylene (PE), polypropylene (PP), polystyrene (PS), polyvinyl chloride (PVC), polyethylene terephthalate (PET), polycarbonate (PC), poly (methyl methacrylate) (PMMA), and polyurethane (PU)—blended with various performance-enhancing additives, including stabilizers, flame retardants, plasticizers, fillers, and pigments [2]. However, the ubiquity of these materials has led to a massive accumulation of plastic waste generated by diverse human activities, ranging from industrial manufacturing to the routine use of consumer cleaning products, cosmetics, and medical supplies [3].

Once released into the environment, larger plastic pieces undergo various degradation processes—including photodegradation, oxidation, hydrolytic degradation, and biodegradation [4]. The rate and extent of this biodegradability are heavily governed by the raw material type, the polymer’s inherent chemical composition and crystalline structure, and ambient environmental conditions like sunlight, water, wind, and biological activity [5,6,7]. Over time, this fragmentation yields debris across a broad spectrum of sizes: megaplastics (>50 cm), macroplastics (5–50 cm), mesoplastics (0.5–5 cm), MPs (<5 mm), and nanoplastics (≤0.1 μm) [4,8]. The concept of MPs was first introduced in 2004 by Thompson et al. at the University of Plymouth [8]. Today, MPs are broadly classified into two categories: primary particles, which are intentionally manufactured at a small scale (e.g., pre-production pellets, industrial abrasives, and cosmetic microbeads), and secondary particles, which derive predominantly from the UV-induced and mechanical weathering of larger plastic items.

MPs enter the biosphere through numerous pathways, such as the shedding of microfibers from synthetic textiles during washing, the erosion of synthetic rubber tires, and industrial discharge. Following environmental transport and migration, these particles inevitably infiltrate animals and plants, ultimately entering the human body via inhalation, skin contact, and ingestion. Dietary ingestion represents a highly prevalent route of exposure, with MPs frequently detected in common food and beverages, including seafood, drinking water, and beer [9]. Exposure models in mice have already demonstrated that ingested MPs accumulate significantly in the stomach, intestines, liver, and other major organs [10,11].

Because MPs possess high corrosion resistance, they persist when entering the digestive tract. Exposure to digestive fluids alters their surface roughness and particle size, making them more stable within the digestive lining and highly prone to adsorbing external toxins [12]. Consequently, their unique surface properties allow them to act as ideal vectors for various environmental contaminants, including organic pollutants, heavy metals, and pathogenic microorganisms, forming contaminant–microplastic complexes with severe potential transmission risks [13,14,15].

Crucially, physiological tissue barriers do not completely prevent the invasion of these particles. Evidence from experimental models suggests that the behavior of plastic particles at the gastrointestinal–epithelial interface depends on several particle-related characteristics, including their dimensions, morphology, and surface properties, as well as on the condition of the epithelial barrier. Investigations have covered a broad range of particle sizes and experimental settings. In vivo experiments involving oral exposure in mice have examined 5 µm polystyrene particles, while ex vivo studies using porcine and human intestinal tissues have evaluated particles of 0.05, 1, and 10 µm. Taken together, these findings show that epithelial interactions and potential uptake vary according to both particle size and the experimental system. Evidence specifically demonstrating passage through an intact human gastric epithelium, however, is still scarce. Thus, results obtained in animal or ex vivo models should be interpreted cautiously when considering their applicability to humans [16,17,18]. Once inside, ingested MPs can induce acute and chronic gastric inflammation, disrupt the gut barrier, and trigger severe dysbiosis. For instance, exposure to PMMA MPs triggers senescence in gastric mucosal epithelial cells through the overproduction of reactive oxygen species (ROS), the release of pro-inflammatory mediators, and the impairment of DNA repair via the inhibition of the non-homologous end joining (NHEJ) pathway in a concentration-dependent manner [19]. Similarly, PE MPs have been shown to cause hypertrophy and hyperplasia in the gastric mucosa, while orally administered PS MPs can integrate directly into the tissue matrices of the stomach, liver, and kidneys [20,21].

While the evidence linking MPs to gastrointestinal distress is steadily expanding, the current literature remains highly asymmetrical, focusing predominantly on lower intestinal dysbiosis and colorectal presence. Consequently, the immediate, upstream consequences of MP exposure within the human stomach remain poorly understood and significantly understudied.

Furthermore, a critical gap exists in clinical and experimental translation: to date, no prominent studies have established a direct correlation between the physical presence of MPs in harvested gastric tissue and systemic variations in standard biochemical blood profiles. Investigating whether localized gastric plastic infiltration mirrors or drives alterations in routine metabolic, inflammatory, or hepatic biochemical markers represents an unexplored frontier in environmental toxicology.

In view of these limitations, the present study investigates the occurrence and characteristics of suspected MP-like particles in human gastric specimens collected at autopsy. Polarized light microscopy was included as a complementary approach to examine birefringent structures and document their morphological features and anatomical location within gastric tissue. The study further explores whether particle detection is associated with local histopathological alterations and routine biochemical parameters. By combining microscopic observations, histopathological evaluation, and clinical laboratory data, this work provides an integrated assessment of suspected MP-like particles in the human stomach and explores their possible biological relevance.

## 2. Materials and Methods

### 2.1. Study Population and Ethical Approval

This retrospective post-mortem study evaluated tissue specimens harvested from 55 adult individuals of both sexes, aged between 21 and 94 years. The study cohort comprised cases registered at the Sibiu County Emergency Clinical Hospital, Romania, between March 2022 and December 2025. Tissue samples from the stomach were systematically collected during routine autopsy procedures.

The study protocol was reviewed and formally approved by the Ethics Committee of Lucian Blaga University of Sibiu (No. 10/20 July 2021). Prior to tissue harvesting, written informed consent for the use of post-mortem specimens and the analysis of associated clinical data was obtained from the next of kin for all included cases, in strict accordance with the Declaration of Helsinki.

### 2.2. Data Collection and Variable Definitions

Clinical, demographic, and laboratory parameters were retrospectively retrieved from institutional electronic medical records. For each subject, baseline demographic and clinical characteristics were systematically documented, including age, sex, area of residence (urban or rural), the presence of underlying neoplastic disease, and any prior history of exposure to radiotherapy, chemotherapy, or both. Laboratory data included the most recent biological results available before death. These included blood glucose, liver biomarkers (alanine aminotransferase—ALT, aspartate aminotransferase—AST), pancreatic biomarkers (amylase, lipase), renal function parameters (creatinine, urea, eGFR), electrolytes (sodium, potassium, chloride), inflammatory biomarkers (C-reactive protein—CRP, procalcitonin), and hematological indices (leucocytes, erythrocytes, platelets, hemoglobin, hematocrit). Additionally, key cardiovascular and pulmonary comorbidities of interest—specifically atherosclerosis, chronic ischemic heart disease, myocardial infarction, and bronchopneumonia—were extracted from the diagnostic history.

### 2.3. Sample Collection for Digestion

For microplastic analysis, 5 g of gastric tissue was collected in sterile 50 mL tubes with aluminum caps. To minimize the risk of external contamination, the use of plastic instruments or any tools containing plastic components was strictly avoided throughout the sampling procedure. The collected samples were stored at −80 °C prior to analysis.

### 2.4. Sample Collection for Histological Analysis

Gastric tissue samples were collected within 24 h post-mortem from individuals undergoing autopsy to determine the cause of death. Following dissection, three tissue fragments (approximately 1 cm^3^ each) were harvested from the region of interest and fixed in 30 mL of Davidson’s solution within 50 mL capped tubes. The Davidson solution was prepared using 2% formaldehyde solution (37–40%), 35% absolute ethanol, 10% glacial acetic acid, and 53% distilled water [22]. To prevent external contamination, only non-plastic instruments were used during collection. Each sample was labeled to maintain traceability and enable correlation with the corresponding medical records.

### 2.5. Detection and Quantification of Suspected MP-like Particles

The analytical procedure was based on the protocol previously applied by our research group [23], with application to gastric tissue specimens in the present study. To minimize contamination, laboratory equipment was cleaned with acetone before use, personnel wore cotton laboratory coats, and sample processing was performed in a microbiological hood in an isolated environment without air circulation.

For each case, 1 g of gastric tissue was placed in a 50 mL glass tube and treated with 20 mL of 30% hydrogen peroxide (H_2_O_2_), while the remaining tissue was retained for potential repeat analyses. The tubes were covered with aluminum foil and incubated at 40 °C for seven days with periodic vortexing. After digestion, samples were cooled to room temperature and centrifuged at 2000 rpm for 2 min. The supernatant was vacuum-filtered through 1 μm glass-fiber filters, which were subsequently dried at 40 °C, treated with an additional 3 mL of H_2_O_2_, rinsed with 40 mL of ultrapure water, and oven-dried.

For fluorescence-based visualization, approximately 1 mL of Nile Red solution (0.001 mg/mL in absolute ethanol) was applied to the filter surface. After 15 min at room temperature, the filters were rinsed with 60 mL of ultrapure water and dried overnight. Examination was performed using a transilluminator, and images were acquired at ISO 200, F6.3 aperture, 1/2 s exposure time, and 45 mm focal length.

Quality-control procedures were included in each analytical batch. A reagent blank underwent the same digestion, filtration, staining, imaging, and image-analysis procedure as the tissue samples. No suspected particles were detected in the blanks; therefore, blank correction was not required. Recovery was evaluated using three controls, each spiked with ten artificially generated ABS particles before processing. Recoveries were 80%, 90%, and 110%, respectively, with a mean recovery of 93.3%; the minFeret of the ABS particles ranged from 112.74 to 863.65 µm. The value exceeding 100% may reflect particle fragmentation during processing together with counting variability associated with low particle numbers.

Particle quantification was performed using ImageJ (version 1.54p), following the methodological approach described by Curtean-Bănăduc et al. (2023) [24]. Because 1 g of tissue was processed for each case, particle burden was reported as suspected MP-like particles per gram of gastric tissue (particles/g). The entire filter surface was examined, and candidate particles were initially identified according to their morphology and fluorescence characteristics. ImageJ-detected structures were subsequently reviewed manually to exclude Nile Red residues and other nonspecific fluorescent artifacts.

An operational minFeret threshold of 56.21 µm (0.05621 mm), corresponding to a minimum accepted dimension of three pixels after spatial calibration, was used to reduce background-related detections. Structures below this threshold were excluded, as were structures immediately above it when their morphology or optical appearance was consistent with background artifacts. This value represents an operational threshold for the present image-analysis workflow rather than a polymer-specific analytical detection limit.

Structures observed independently in histological sections were characterized according to their morphology, dimensions, optical properties, and anatomical location and were not included in the digestion-derived particle counts. As the analytical approach was based on Nile Red fluorescence, morphological assessment, and image analysis without polymer-specific spectroscopic confirmation, all detected structures are referred to as suspected MP-like particles. Definitive polymer identification would require complementary techniques such as µ-FTIR, Raman spectroscopy, or pyrolysis–gas chromatography/mass spectrometry.

### 2.6. Histological Assessment

To evaluate structural changes in the gastric tissue—specifically inflammation, fibrosis, and necrosis—sections were stained with hematoxylin and eosin (H&E), the histopathological gold standard [25]. These histological slides were first examined under unpolarized light using a Leica DM3000 microscope. Tissue sections were additionally examined by polarized light microscopy to identify and morphologically characterize birefringent structures of possible exogenous origin [26]. Polarized light microscopy was performed using a Leica DM2500 microscope equipped with polarizing filters and an integrated camera for image acquisition. Polarized light microscopy was used as a complementary morphological screening method to visualize birefringent structures in histological sections. Since birefringence may originate from both plastic and non-plastic materials, observations were limited to particle morphology, optical features, and tissue location, without assigning or confirming polymer identity.

### 2.7. Suspected MP-like Particle Size Estimation

The size of the identified suspected MP-like particles was determined via image analysis using ImageJ software, following a methodology based on Curtean-Bănăduc et al. (2023) [24], and coupled with polarized light microscopy. Measurements were performed on the acquired microscopic images after calibrating the spatial scale, with the final values expressed in micrometers (µm).

### 2.8. Statistical Analysis

Statistical analysis was performed using IBM SPSS Statistics, version 26.0 (IBM Corp., Armonk, NY, USA). Continuous variables are presented as mean ± standard deviation or median with interquartile range, according to data distribution, while categorical variables are reported as frequencies and percentages. Comparisons between individuals with and without detectable suspected gastric MP-like particles were performed using an independent-samples *t*-test or the Mann–Whitney U test for continuous variables and the chi-square or Fisher’s exact test for categorical variables, as appropriate. Associations with histopathological findings and clinical diagnoses were assessed using Fisher’s exact test.

All tests were two-sided, with statistical significance set at *p* < 0.05.

## 3. Results

### 3.1. Study Population Characteristics According to Suspected Gastric MP-like Particle Detection Status

As shown in Table 1, suspected gastric MP-like particles were detected in 15 of the 55 individuals (27.3%), whereas 40 individuals (72.7%) had no detectable particles. No statistically significant differences were observed between individuals with and without detectable suspected gastric MP-like particles regarding demographic characteristics, including age, sex, and area of residence (all *p* > 0.05). Similarly, the prevalence of neoplasms and previous radiotherapy and/or chemotherapy did not differ significantly between the two groups.

No significant between-group differences were identified for blood glucose or for liver, pancreatic, renal, inflammatory, and hematological biomarkers (all *p* > 0.05). Individuals with detectable suspected gastric MP-like particles showed numerically higher serum chloride levels than those without detectable particles (106.32 ± 8.63 vs. 102.30 ± 6.93 mmol/L), although this difference did not reach statistical significance (*p* = 0.079). The remaining electrolyte parameters, including sodium and potassium, were comparable between groups.

### 3.2. Detection Frequency and Burden of Suspected Gastric MP-like Particles

**Figure 1:**
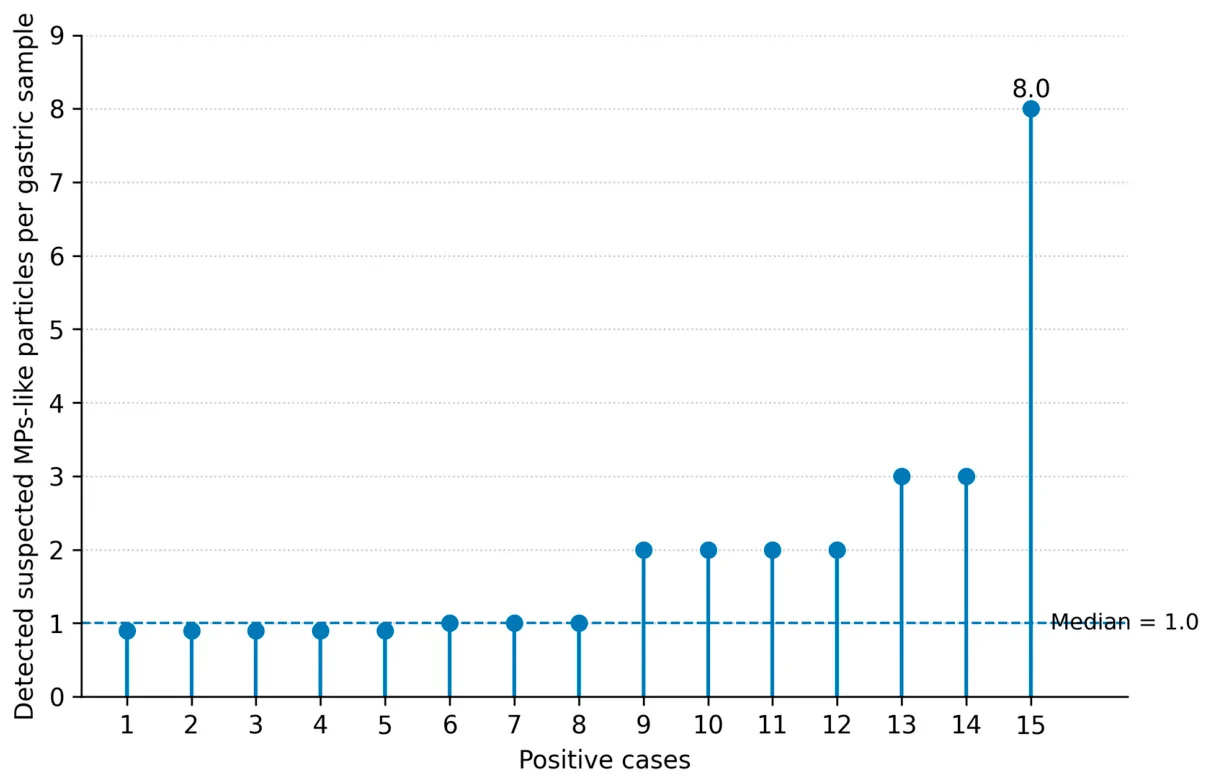
Distribution of suspected MP-like particle counts in positive gastric tissue samples, expressed as particles/g.

Figure 1 illustrates the distribution of suspected gastric MP-like particle burden among the 15 individuals with detectable particles. Most suspected MP-like particle-positive cases had a low particle burden, with eight cases presenting one particle/g. Four additional cases had two particles/g, while two cases had three particles/g. One individual showed a markedly higher burden, with eight particles/g. The median gastric suspected MP-like particle burden was 1.0 particle/g, indicating a right-skewed distribution driven by a single case with a substantially higher particle count.

### 3.3. Gastric Histopathological Alterations According to Suspected MP-like Particle Detection

Histopathological examination identified gastric inflammation in 29 of the 55 individuals (52.7%). Suspected MP-like particles were detected in 15 of these 29 cases, while the remaining 14 were particle-negative. In contrast, none of the 26 individuals without gastric inflammation showed suspected MP-like particles. Thus, inflammation occurred in 100% of particle-positive cases compared with 35.0% of particle-negative cases, demonstrating a statistically significant association (Fisher’s exact test, *p* < 0.001). Given the cross-sectional design, this finding indicates an association but does not allow conclusions regarding its temporal sequence or underlying mechanism.

Necrosis was identified in a single case, which was also positive for suspected MP-like particles; however, no statistically significant relationship was observed (Fisher’s exact test, *p* = 0.273). Fibrotic changes were found in two individuals, both belonging to the particle-positive group (13.3%), although this association was likewise not statistically significant (Fisher’s exact test, *p* = 0.071). Because necrosis and fibrosis were uncommon in this cohort, these findings should be interpreted cautiously.

### 3.4. Additional Clinical Diagnoses According to Gastric Suspected MP-like Particle Detection Status

No statistically significant associations were observed between gastric suspected MP-like particle detection and the additional clinical diagnoses evaluated (Table 2). Although chronic ischemic heart disease tended to be less frequent and bronchopneumonia more frequent among individuals with detectable gastric suspected MP-like particles, these differences did not reach statistical significance. Myocardial infarction showed an identical prevalence in both groups. Overall, the presence of gastric suspected MP-like particles was not associated with the comorbidities investigated.

### 3.5. Microscopic Visualization of Suspected Gastric MP-like Particles Under Polarized Light

**Figure 2:**
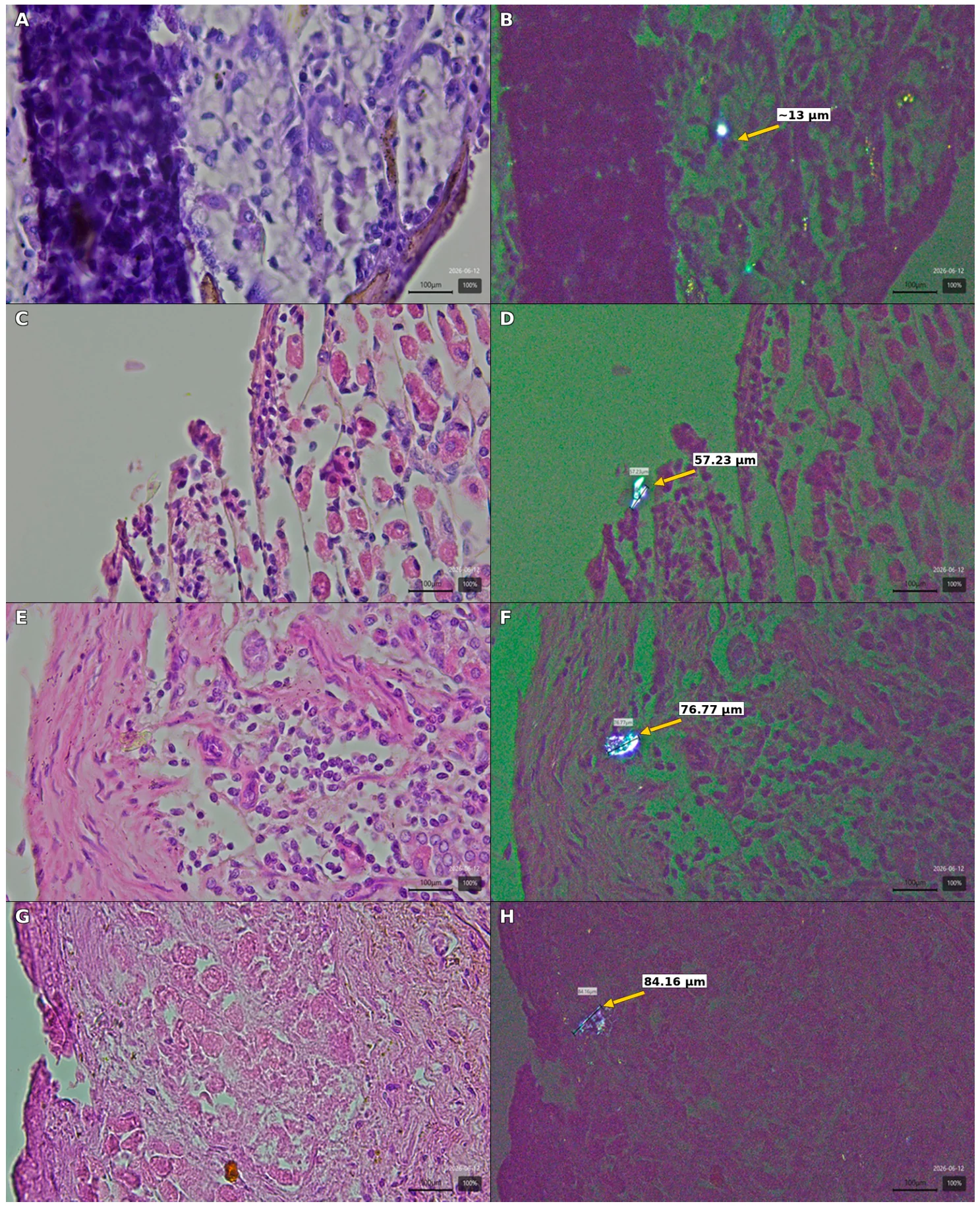
Representative unpolarized and polarized light microscopy images of gastric tissue. (**A**,**B**) Case 5: unpolarized and corresponding polarized light microscopy images. (**C**,**D**) Case 7: unpolarized and corresponding polarized light microscopy images. (**E**,**F**) Case 12: unpolarized and corresponding polarized light microscopy images. (**G**,**H**) Case 22: unpolarized and corresponding polarized light microscopy images. Magnification: 40×.

Representative gastric sections were evaluated for suspected MP-like particles using polarized light microscopy as a complementary visual tool. Exogenous particle detection was performed by analyzing paired polarized and unpolarized images from four selected cases (Figure 2). Three of the structures identified in the histological sections could be measured, with maximum dimensions of 26.52, 52.23, and 84.16 µm. A fourth structure had an irregular appearance, but its size could not be reliably established from the corresponding microscopic image and was therefore considered non-quantifiable. Morphological examination showed variable appearances, including hexagonal, irregular, and fiber-like structures. Their anatomical distribution involved both the gastric mucosa and the gastric lumen.

In Case 5, a regular, bright, hexagonal structure with a maximum diameter of 26.52 micrometers was observed within the mucosa. Its regular appearance could suggest the presence of an exogenous suspected MP-like particle.

In Case 7, a bright fibrillar structure with green and violet iridescence, measuring 52.23 micrometers at its largest dimension, was observed within the gastric lumen. Its appearance and coloration could suggest the presence of an exogenous suspected MP-like particle.

Polarized light examination revealed distinct birefringent structures in Cases 12 and 22. In Case 12, a brightly birefringent, angular structure showing violet-greenish optical features was located in the mucosa above the muscularis mucosae. Case 22 showed a fibrillar structure with irregular morphology and violet-greenish birefringence in the gastric mucosa, measuring 84.16 µm at its greatest dimension. Based on their appearance and localization, these findings were classified as suspected MP-like particles of possible exogenous origin. However, their chemical identity remains undetermined because no polymer-specific spectroscopic analysis was undertaken. Accordingly, the morphological and dimensional characteristics reported here represent screening observations and do not establish that the detected structures are composed of plastic.

## 4. Discussion

The present exploratory study identified suspected MP-like particles in human gastric tissue and found a significant relationship between their detection and local gastric inflammation. In contrast, particle-positive and particle-negative cases did not differ significantly in the evaluated systemic biochemical parameters, including CRP, procalcitonin, complete blood count, ALT, AST, urea, and creatinine. Histological examination nevertheless showed that inflammatory changes frequently accompanied the presence of suspected particles. In selected particle-positive cases, polarized light microscopy was additionally used to examine the corresponding histological sections and characterize birefringent structures within the tissue.

Suspected MP-like particles were detected in 15 of 55 cases (27.3%), adding to the emerging evidence that environmentally derived particles may reach human tissues following chronic exposure. The digestion and filtration procedure adapted from Curtean-Bănăduc et al. enabled subsequent Nile Red-based fluorescence screening while limiting interference from residual biological material [24]. Histopathologically, gastric inflammation was present in every particle-positive case and represented the predominant tissue alteration, whereas fibrosis was observed in two cases and necrosis in one. Taken together, these observations indicate an association between suspected particle detection and gastric inflammation. However, this relationship should not be interpreted as causal, particularly because other determinants of gastric inflammation, including Helicobacter pylori infection, medication use, alcohol consumption, smoking, and psychological stress, were not comprehensively assessed in the present study [25]. These unmeasured factors therefore remain potential confounders when interpreting the observed association.

Polarized light examination identified birefringent structures with an exogenous particle-like appearance in four representative cases. Three were located within the gastric mucosa, whereas one was identified in the gastric lumen. These two locations have different interpretative implications: a luminal particle may reflect recently ingested or transient material and cannot be considered evidence of tissue incorporation, while the presence of a structure within the mucosa may indicate closer contact with gastric tissue. However, the post-mortem cross-sectional design does not allow reconstruction of the timing or pathway by which these structures reached their observed locations, nor can it determine whether mucosal localization preceded or followed the development of histopathological changes. Importantly, identification was based exclusively on optical and morphological features. Thus, the observations remain presumptive in the absence of polymer-specific characterization by techniques such as µ-FTIR or µ-Raman spectroscopy. Overall, the birefringent appearance and anatomical distribution are compatible with tissue-associated exogenous particle-like structures, but these findings should be regarded as supportive rather than definitive evidence of microplastics [26].

The biological likelihood of the association between MPs and gastric inflammation is supported by experimental evidence. Following ingestion, MPs transit through the oral cavity and esophagus into the stomach, where their retention time is highly dependent on the food matrix; while liquids exhibit rapid clearance, solid foods typically prolong gastric emptying to a window of 2 to 5 h [27]. During this residence phase, the ingested particulates are exposed to a highly acidic gastric environment (pH: 1.5–3.5), which undergoes a dynamic digestive process, including fragmentation, oxidation, and the adsorption of microorganisms, and other environmental contaminants. These changes increase particle surface area and chemical reactivity, enhancing interactions with the gastric mucus. Rather than remaining biologically inert, MPs undergo continuous transformation within the gastrointestinal tract, potentially increasing their capacity to interact with host tissues and elicit adverse biological responses.

Experimental studies demonstrate that MPs do not remain inert within the digestive tract. Instead, the gastrointestinal environment actively alters their structural morphology, while the generation of secondary micro- and nano-fragments substantially expands their specific contact surface area [28]. Concurrently, robust gastric motility drives intense physical contact between these digestively altered MPs and the superficial mucus layer.

Upon interaction, the plastic particles become entrapped within the mucin network, triggering a significant decrease in mucus viscosity. This structural thinning severely compromises the rheological and protective capabilities of the mucosal barrier, accelerating the diffusion of both the polymer fragments and co-ingested pathogens through the fluidic mucus. By facilitating migration toward the epithelial surface, this disruption increases the likelihood of epithelial injury, a process further exacerbated by gastric motility promoting repeated physical interactions and prolonging local exposure [29].

Depending on their distinct size profiles, the retained MPs may subsequently breach the gastric lining and penetrate the underlying tissues. Given these size dynamics, paracellular infiltration via persorption stands out as a highly applicable entry mechanism [30]. During this process, physiological contractile forces mechanically push larger particles through transient intercellular gaps that naturally form during normal cell extrusion and epithelial desquamation. Ultimately, experimental evidence from human gastric cell lines confirms that micro- and nanoplastics systematically impair the stomach’s physiological defenses—disrupting epithelial barrier integrity, inhibiting gastric juice secretion, and suppressing localized antioxidant capacity [31].

At the cellular level, the cytotoxicity of these particles is strictly dictated by their physicochemical properties, including size, morphology, polymer composition, and weathering state. Generally, smaller, highly weathered particles exhibit enhanced cellular internalization, heightened induction of oxidative stress, and more severe mitochondrial dysfunction [32,33,34,35]. This cellular damage establishes a vicious pathological cycle, wherein sustained oxidative stress and chronic inflammation reciprocally amplify mitochondrial degradation [36,37,38]. Critically, MPs may also serve as physical vectors for *H. pylori* within the gastric environment; recent data indicate that the co-presence of MPs and *H. pylori* synergistically amplifies the localized mucosal inflammatory response, compounding the overall risk of gastric pathology [10].

Interestingly, despite the clear histopathological association with gastric inflammation, no significant differences were observed between suspected MP-like particle-positive and suspected MP-like particle-negative individuals regarding usual biochemical parameters, inflammatory markers, liver enzymes, renal function tests, pancreatic enzymes, or hematological parameters. Several explanations may account for these findings. First, the inflammatory response associated with suspected MP-like particles may remain localized within the gastric mucosa without inducing measurable systemic effects. Second, the relatively low particle burden observed in most positive cases may be insufficient to alter circulating biomarkers. Finally, the heterogenous clinical characteristics of an autopsy population, including multiple underlying diseases and causes of death, may obscure subtle systemic changes attributable to MP exposure. These observations suggest that tissue-level histopathological examination may be more sensitive than routine laboratory testing for detecting the biological consequences of gastric suspected MP-like particle presence.

The present study has several important strengths. It integrates complementary analytical methodologies—including chemical digestion, digital particle quantification, conventional histopathology, and polarized light microscopy—while implementing contamination-control measures across the entire sample-processing workflow. This multimodal approach increases confidence in the detection of suspected MP-like particle structures and enables simultaneous evaluation of tissue pathology. Moreover, correlating particle detection with histopathological findings and clinical laboratory parameters provides a broader assessment of the potential biological significance of gastric suspected MP-like particle occurrence beyond particle identification alone.

Nevertheless, several limitations should be acknowledged. The relatively small sample size and single-center retrospective design limit statistical power and the generalizability of the findings. Information regarding individual environmental exposure, occupational history, dietary habits, smoking, alcohol consumption, medication use, and *Helicobacter pylori* infection was not available, preventing adjustment for important confounding variables. Accordingly, the observed relationship cannot be interpreted as demonstrating a causal effect of suspected MP-like particles on gastric inflammation. Furthermore, although polarized light microscopy represents a valuable screening tool, it cannot definitively identify polymer composition; for this reason, all detected particles should be interpreted as suspected MP-like particles unless their polymeric nature is verified using polymer-specific techniques. Further clarification of the mechanisms through which MPs may affect the human digestive system will require advanced monitoring strategies. For instance, labeling MPs would allow their movement along the food chain and into the human body to be monitored, while analysis of body fluid samples could contribute to mapping their systemic distribution [39].

## 5. Conclusions

Overall, the present findings provide further evidence of suspected MP-like structures in human gastric tissue and their association with localized inflammatory changes. No significant differences in systemic biochemical parameters were observed according to particle detection status. The co-occurrence of suspected MP-like structures with histopathological alterations warrants further investigation into a potential relationship between environmental particle exposure and gastric mucosal changes. However, the screening-based identification of these structures, together with the inability to account for relevant confounding factors or establish the temporal sequence of exposure and tissue alterations, precludes causal interpretation. Accordingly, these observations should be regarded as exploratory and hypothesis-generating.

Further studies combining histopathological assessment with polymer-specific analytical techniques are needed to characterize these particles and clarify the potential implications of chronic environmental plastic exposure for gastrointestinal health.

## Figures and Tables

**Table 1:** Demographic, clinical, and laboratory characteristics according to suspected gastric MP-like particle detection status.

Variable	Overall (*n* = 55)	Suspected MP-like Particles Absent (*n* = 40)	Suspected MP-like Particles Present (*n* = 15)	*p*-Value
Demographic characteristics				
Age, years	68.62 ± 15.35	69.10 ± 13.62	67.33 ± 19.75	0.708
Male sex, *n* (%)	29 (52.7)	22 (55.0)	7 (46.7)	0.581
Urban residence, *n* (%)	34 (61.8)	26 (65.0)	8 (53.3)	0.428
Clinical characteristics				
Neoplasm, *n* (%)	14 (25.5)	12 (30.0)	2 (13.3)	0.206
Radiotherapy and/or chemotherapy, *n* (%)	8 (14.5)	6 (15.0)	2 (13.3)	0.876
Blood glucose, mg/dL	132.00 (102.00, 194.00)	144.50 (102.00, 207.00)	118.00 (98.50, 147.00)	0.379
Liver biomarkers				
DBIL, mg/dL	0.94 (0.81, 1.30)	0.94 (0.78, 1.30)	0.96 (0.86, 1.59)	0.776
TBIL, mg/dL	1.70 (1.35, 2.57)	1.70 (1.43, 2.58)	1.94 (0.80, 2.56)	0.917
AST, U/L	43.00 (30.50, 212.00)	50.00 (33.00, 253.00)	37.00 (30.00, 171.00)	0.791
ALT, U/L	31.00 (19.00, 129.00)	37.00 (24.00, 183.00)	24.50 (15.00, 78.00)	0.564
Pancreatic biomarkers				
Amylase, U/L	44.00 (33.00, 82.00)	58.00 (18.00, 90.00)	38.00 (33.00, 46.00)	0.490
Lipase, U/L	25.00 (15.00, 105.00)	25.00 (16.00, 81.50)	22.00 (11.00, 105.00)	0.820
Renal biomarkers				
Urea, mg/dL	54.00 (40.50, 89.00)	49.50 (40.50, 81.50)	69.00 (44.00, 109.00)	0.364
Creatinine, mg/dL	1.26 (0.80, 2.06)	1.14 (0.94, 2.20)	1.40 (0.80, 1.65)	0.671
eGFR, mL/min/1.73 m^2^	58.00 (29.00, 86.00)	58.00 (26.00, 92.00)	53.00 (31.00, 82.00)	0.671
Electrolytes				
Sodium, mmol/L	139.67 ± 7.24	138.85 ± 6.99	141.87 ± 7.69	0.171
Potassium, mmol/L	4.29 ± 0.87	4.38 ± 0.88	4.04 ± 0.83	0.201
Chloride, mmol/L	103.40 ± 7.56	102.30 ± 6.93	106.32 ± 8.63	0.079
Inflammatory biomarkers				
CRP, mg/L	153.46 (43.43, 206.68)	153.46 (43.43, 206.68)	124.86 (35.97, 220.94)	0.205
Procalcitonin, ng/mL	1.58 (0.32, 33.39)	1.18 (0.29, 33.39)	1.99 (0.87, 28.67)	0.635
Hematological biomarkers				
Leukocytes, ×10^3^/µL	12.64 (8.03, 17.71)	13.68 (7.60, 17.81)	11.11 (8.49, 15.85)	0.637
Erythrocytes, ×10^6^/µL	3.93 ± 1.05	4.01 ± 1.10	3.72 ± 0.90	0.365
Platelets, ×10^3^/µL	213.00 (132.00, 302.00)	209.00 (108.50, 313.00)	220.00 (181.00, 293.00)	0.545
Hemoglobin, g/dL	11.80 ± 3.09	12.01 ± 3.22	11.25 ± 2.74	0.420
Hematocrit, %	36.00 ± 9.29	36.65 ± 9.82	34.30 ± 7.73	0.409

Note: Data are presented as mean ± standard deviation, median (interquartile range), or *n* (%), as appropriate; MPs, microplastics; DBIL, direct bilirubin; TBIL, total bilirubin; AST, aspartate aminotransferase; ALT, alanine aminotransferase; eGFR, estimated glomerular filtration rate; CRP, C-reactive protein. A *p*-value < 0.05 was considered statistically significant.

**Table 2:** Additional clinical diagnoses according to gastric suspected MP-like particle detection status.

Diagnosis	Overall	Suspected MP-like Particles Absent	Suspected MP-like Particles Present	*p*-Value
Atherosclerosis	18 (32.7%)	14 (35.0%)	4 (26.7%)	0.749
Chronic ischemic heart disease	37 (67.3%)	29 (72.5%)	8 (53.3%)	0.208
Myocardial infarction	11 (20.0%)	8 (20.0%)	3 (20.0%)	1
Bronchopneumonia	24 (43.6%)	16 (40.0%)	8 (53.3%)	0.543

Note: Data are presented as *n* (%). Comparisons between groups were performed using Fisher’s exact test. MPs, microplastics. A *p*-value < 0.05 was considered statistically significant.

## Data Availability

The original contributions presented in this study are included in the article. Further inquiries can be directed to the corresponding authors.
